# Vascular Complication Following Injection of Particulated Human Acellular Dermal Matrix: A Case Report

**DOI:** 10.1111/jocd.71049

**Published:** 2026-07-14

**Authors:** Sin Woo Sung, So Young Lee, Joon Seok, Beom Joon Kim

**Affiliations:** ^1^ Department of Dermatology Chung‐Ang University College of Medicine Seoul Korea

To the Editor,

1

With the increasing demand for anti‐aging interventions, the clinical use of injectable fillers has continued to expand. Although filler‐related vascular complications are rare, they may result in skin necrosis, stroke, or blindness [1]. Recently, injectable particulated human acellular dermal matrix (phADM), an extracellular matrix (ECM)‐based biomaterial derived from allogenic human skin, has been developed and attempted for volume augmentation and improvement of skin quality. In a randomized split‐face clinical trial, phADM improved skin density, volume, and skin quality without serious adverse events [2]. However, evidence regarding the vascular safety profile of phADM remains limited. Herein, we present a case of vascular complication following phADM injection, highlighting the potential risk of ischaemia associated with this emerging biomaterial.

A 44‐year‐old Caucasian female presented with a reticulated erythematous patch with yellow pustules and crusts on the forehead (Figure 1A). Three days prior, she had received injections of phADM (Elravie Re2O; L&C BIO, Seongnam, Korea) into the forehead and both cheeks at a local clinic. The product had been hydrated with 2 mL of normal saline and 1 mL of non‐cross‐linked HA. She reported no subjective symptoms after the procedure and was discharged. However, 2 days after injection, she developed pain, pruritus, erythema, and pustules on the forehead. On the day of symptom onset, 1250 units of hyaluronidase were injected into the affected area at the local clinic. Despite the treatment, the symptoms persisted and she was referred to our clinic the following day. Based on the lesion distribution, vascular compromise involving the left supratrochlear artery territory was suspected. Pus microscopy, culture and sensitivities were negative, and laboratory evaluation revealed mild leukocytosis, while other inflammatory markers were within normal limits. She was treated with hyperbaric oxygen therapy, local wound care, and empirical medications including enteric‐coated aspirin 100 mg and vasodilators (sublingual nitroglycerin 0.6 mg and oral limaprost 5 μg). One day later, no new pustules were observed, and she reported symptomatic improvement (Figure 1B). She was subsequently lost to follow‐up. At 9 weeks, a patient‐provided photograph demonstrated resolution of erythema and pustules with residual atrophic scarring (Figure 1C).

**FIGURE 1:**
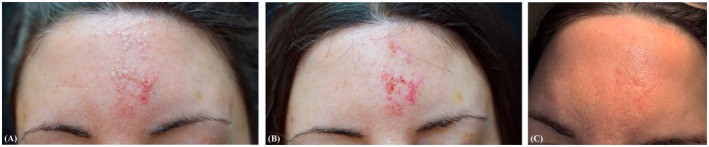
Clinical photographs of the patient's forehead. (A) Photograph taken 3 days after phADM injection, showing a reticulated erythematous patch with multiple yellow pustules on the forehead and focal areas of erosion. She had received hyaluronidase injection into the affected area the previous day. (B) Photograph taken 4 days after injection. Following hyperbaric oxygen therapy, local wound care and empirical medications including aspirin and vasodilators, no newly developed pustules were observed. (C) Patient‐provided photograph obtained 9 weeks after treatment. Resolution of erythema and pustules is noted, with residual atrophic scarring.

Unlike typical filler‐induced vascular complications, which present with immediate blanching and pain during or shortly after injection, she remained asymptomatic for 2 days before ischemic changes. Although 1 mL of non‐cross‐linked HA was used for hydration, it is rapidly degraded and cleared from the intravascular space within 24 h [3]. Therefore, non‐cross‐linked HA may not have been the primary cause of ischemia. To our knowledge, the intravascular behavior of injectable phADM has not been characterized. In this case, phADM may have caused intravascular obstruction of the left supratrochlear artery or its branches. Unlike HA fillers, phADM would not be dissolved by hyaluronidase, potentially making phADM‐associated vascular compromise more difficult to manage. Intravascular phADM may also provide prothrombotic ECM surfaces, as collagen and fibronectin are known to promote platelet adhesion and aggregation [4]. Concurrent endothelial injury and glycocalyx disruption may further remove an antithrombotic barrier, promoting thrombosis and persistent microvascular ischemia [5]. In this context, aspirin was administered empirically to limit thrombotic propagation, although its efficacy remains uncertain. Further studies are needed to elucidate the pathogenesis and management of phADM‐associated vascular complications.

This report has several limitations. Clinical information including the injection technique and product preparation process could not be verified because the procedure was performed at an external clinic. The patient was lost to formal clinical follow‐up after 3 days, and the 9‐week outcome was assessed using a patient‐provided photograph. Nevertheless, this case suggests that injectable phADM is not free from the risk of vascular complications and highlights the need for caution when using this emerging biomaterial.

## Author Contributions

S.W. Sung: writing – original draft preparation, literature review; S.Y. Lee: writing – review and editing; J. Seok: clinical investigation and data acquisition; B.J. Kim: supervising, writing – review and editing.

## Funding

The authors have nothing to report.

## Consent

The patients in this manuscript provided written informed consent for the publication of their case details and clinical pictures.

## Conflicts of Interest

The authors declare no conflicts of interest.

## Data Availability

The data that support the findings of this study are available from the corresponding author upon reasonable request.
